# Draft Genome Sequences of Three Penicillin-Resistant Neisseria gonorrhoeae Strains Isolated in Cincinnati, Ohio, in 1994

**DOI:** 10.1128/MRA.00074-21

**Published:** 2021-03-18

**Authors:** Thomas C. Murphy, Tatum D. Mortimer, Robert A. Nicholas, Crista B. Wadsworth

**Affiliations:** a Rochester Institute of Technology, Thomas H. Gosnell School of Life Sciences, Rochester, New York, USA; b Department of Immunology and Infectious Diseases, Harvard T. H. Chan School of Public Health, Boston, Massachusetts, USA; c Department of Pharmacology, University of North Carolina at Chapel Hill, Chapel Hill, North Carolina, USA; d Department of Microbiology and Immunology, University of North Carolina at Chapel Hill, Chapel Hill, North Carolina, USA; University of Maryland School of Medicine

## Abstract

Here, we report the draft genome sequences of three penicillin-resistant Neisseria gonorrhoeae isolates. We include associated data on MICs and genetic relationships to other N. gonorrhoeae strains collected from across the United States. Resistance mutations known to contribute to reduced penicillin susceptibility are annotated in each genome.

## ANNOUNCEMENT

Penicillin resistance in Neisseria gonorrhoeae can be gained through two mechanisms, inheritance of a plasmid-borne penicillinase (*bla*_TEM-1_) or acquisition of chromosomal mutations. However, the complete suite of causative mutations underlying chromosomal resistance has yet to be determined. Contributors have been identified in a chromosomally mediated resistant N. gonorrhoeae strain (CMRNG), FA6140, isolated from an infected individual in Durham, North Carolina, in 1983 (1) and include (i) mutations in *penA* encoding a penicillin-binding protein 2 (PBP2) with a decreased penicillin acylation rate (2), (ii) a mutation in the *mtrCDE* efflux pump (*mtr*) promoter that increases pump expression (3), and (iii) mutations in the porin P1B allele that decrease porin-mediated influx of penicillin (4). An L421P substitution in PBP1, encoded by *ponA*, also plays a role in decreased susceptibility (5). To contribute to the understanding of mutations that reduce penicillin susceptibility, we sequenced the genomes of three strains with different levels of resistance.

N. gonorrhoeae strains 111, 114, and 151 were isolated from infected individuals in Cincinnati, Ohio, in 1994 and were provided by Joan Knapp at the Centers for Disease Control and Prevention (CDC). Bacteria were cultivated on GC agar base medium supplemented with 1% Kellogg’s solution (6) at 37°C in 5% CO_2_. Susceptibility testing was conducted as previously described (7); all isolates were resistant to penicillin, as determined using the Clinical and Laboratory Standards Institute cutoff of ≥2 μg/ml (8), with MICs from 4 to 32 μg/ml (Table 1).

**TABLE 1:** Strain attributes, genome assembly overview, and identified penicillin resistance determinants^*a*^

Attribute	Data for strain:		
	111	114	151
Collection location	Cincinnati, Ohio	Cincinnati, Ohio	Cincinnati, Ohio
Yr	1994	1994	1994
Total length (bp)	2,089,477	2,092,033	2,196,466
No. of contigs	85	79	89
Coverage (×)	229.26	374.19	445.25
*N*_50_ (bp)	67,041	76,838	72,221
No. of coding domains	2,065	2,057	2,192
No. of tRNAs	50	50	48
GC content (%)	52.67	52.66	52.36
SRA accession no.	SRR13215678	SRR13215677	SRR13215676
GenBank accession no.	JAEEFU000000000.1	JAEEFT000000000.1	JAEEFS000000000.1
PEN MIC (μg/ml)	4	6	32
*penA* mutations	D345a, F504L, A510V, A516G, P551S	D345a, F504L, A510V, A516G, P551S	D345a, F504L, A510V, A516G
*porB* mutations	*porB1b*: G120K, A121D	*porB1b*: G120K, A121D	*porB1a*: WT
*mtrR* mutations	WT	WT	A39T
*mtr* promoter mutations	A-deletion	A-deletion	WT
*ponA* mutations	L421P	L421P	WT
*bla*_TEM_	No	No	Yes

a PEN, penicillin; WT, wild-type allele. All assembly statistics are based on contigs of ≥500 bp.

**FIG 1:**
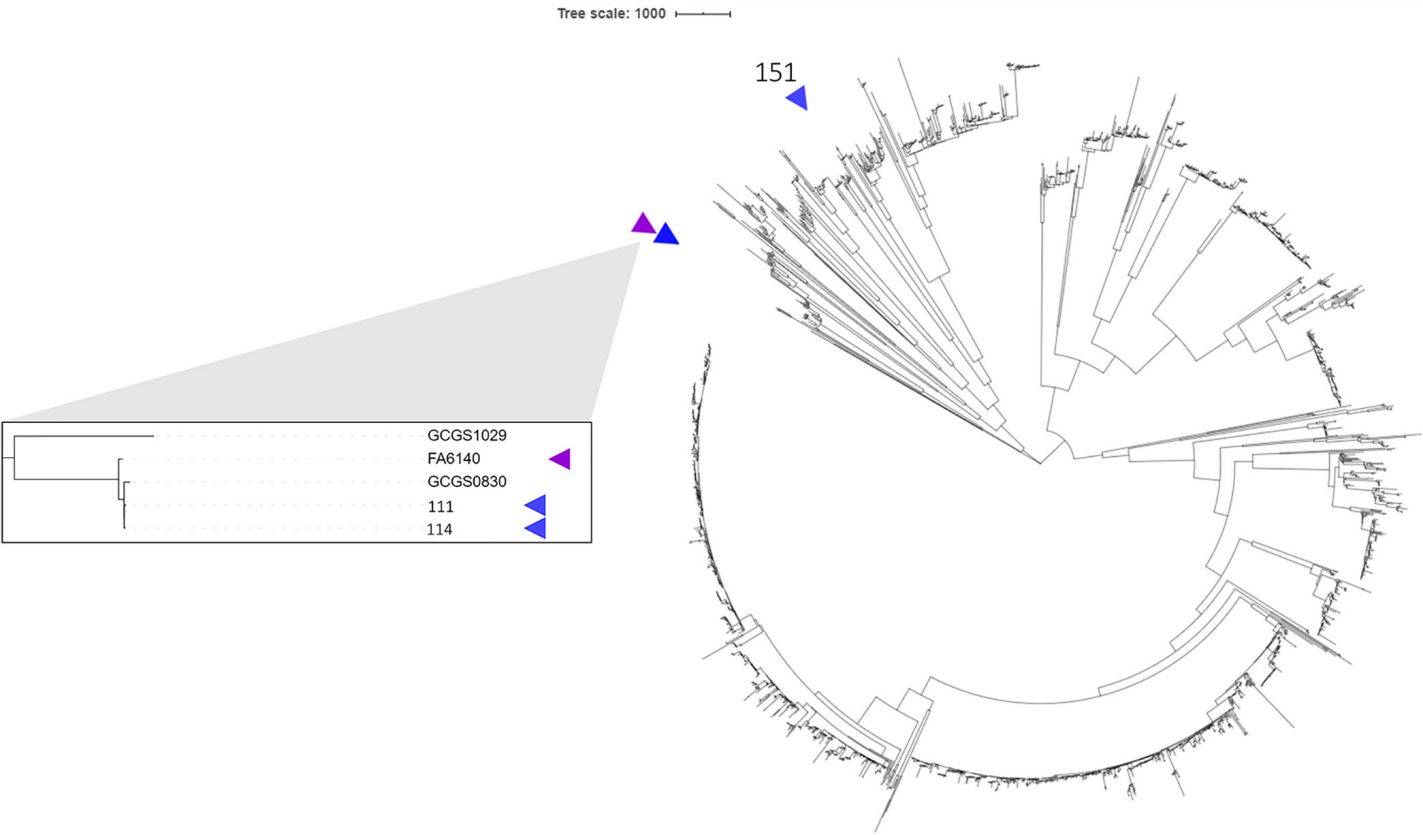
Maximum likelihood whole-genome-based phylogeny of 2,652 N. gonorrhoeae strains collected in the United States between 1983 and 2016. This tree includes 1,102 isolates collected by the Gonococcal Isolate Surveillance Project (GISP) between 2000 and 2013 (13), 649 isolates collected by GISP between 2014 and 2016 (14), 897 isolates collected by the New York City Department of Health and Mental Hygiene between 2011 and 2015 (15), FA6140 (16), and the strains published in this study. Of the newly sequenced strains, 111 and 114 are two of the nearest phylogenetic neighbors to FA6140, a strain that as yet has an unclear mechanism of resistance to penicillin. The purple arrow indicates the position of FA6140 on the tree, and the blue arrows indicate the positions of the strains sequenced in this study.

Cells were grown overnight on agar plates, and genomic DNA was purified using the Thermo Fisher PureLink genomic DNA minikit following lysis in Tris-EDTA buffer with 0.5 mg/ml lysozyme and 3 mg/ml proteinase K. Illumina Nextera XT-prepared libraries were pooled and sequenced using a V3 600-cycle cartridge (2 × 300 bp) on the Illumina MiSeq platform at the Rochester Institute of Technology Genomics Core. For all analyses, default parameters were used except where otherwise noted. Paired-end sequencing resulted in a total of 7.97 million reads across the three samples, and after poor quality sequences were trimmed using Trimmomatic v.0.39 (9), a total of 7.46 million reads remained. SPAdes v.3.13.0 (10) was used for assembly, statistics were reported using QUAST (11), and genes were annotated with Prokka v.1.14.5 (12) (Table 1). We identified resistance mutations by alignment to FA6140 (GenBank accession number CP012027.1) and FA19 (CP012026.1) sequences using BLASTn. To identify the phylogenetic placement of new genomes, we reconstructed the phylogeny of 2,652 gonococcal strains isolated in the United States between 1983 and 2016 (13–16) (Fig. 1). An alignment was created by mapping to NCCP11945 (NC_011035.1) (see reference 17), and Gubbins v.2.4.1 (18), RAxML v.8.2.12 (19), and iTOL v.5 (20) were used to construct and visualize the tree.

Isolates 111 and 114 had penicillin MICs of 4 and 6 μg/ml, respectively, and did not harbor the β-lactamase gene, suggesting that they are similar to the CMRNG strain FA6140. Indeed, out of the 2,652 strains in our cohort, these isolates were among the nearest phylogenetic neighbors to FA6140 (Fig. 1), despite isolation 11 years later from a distinct geographic location. The number of polymorphic sites from the FA6140 reference genome was 484 and 465 for 111 and 114, respectively. These isolates had the same haplotype of known resistance determinants as FA6140 (Table 1).

Strain 151 had a penicillin MIC of 32 μg/ml, and analysis confirmed the presence of *bla*_TEM-1_, indicating that it is a penicillinase-producing strain. The assembly contained a 5,727-bp contig that differed by only two insertions from the top BLAST hit (GenBank accession number MH140435.1), the African-type pJD5 gonococcal plasmid (21). In addition, 151 had an *mtrR* A39T substitution, which increases the expression of *mtrCDE* (22) (Table 1), and substitutions in *penA* (Table 1), but these are likely minor contributors to resistance in this strain.

Future comparative analyses of the CMRNG genomes reported in this announcement may help to further illuminate the genetic basis of penicillin resistance in gonococci.

### Data availability.

The accession numbers for genome assemblies and raw reads are listed in Table 1 and available for download through GenBank and the SRA, respectively. All code is accessible at https://github.com/wadsworthlab.
